# Supplementary material for: A propensity score-matched analysis of mortality in solid organ transplant patients with COVID-19 compared to non-solid organ transplant patients
Source: PLoS One. 2021 Mar 3;16(3):e0247251. doi: 10.1371/journal.pone.0247251 (PMC7928439; doi:10.1371/journal.pone.0247251)
Supplement: Attachment — Submitted filename: Review of the article.docx [file pone.0247251.s002.docx]

These authors investigated the effect of solid organ transplantation (SOT) on COVID-19 mortality. This well-planned article, unfortunately, contains too many errors and mistakes in its writing. I propose a major revision. My concerns were below.

**Introduction**

1. Authors should clearly define the purpose of the study and why these authors conducted this research.

2. As I know that solid organ transplant recipients have more comorbid diseases than patients without SOT. The authors should point to this issue in a separate paragraph in this section.

**Material and method**

1. I recommend authors to use subtitles. Subheadings; patient selection, treatment, outcomes, hospitalization, and ICU admission may use.

2. Inclusion and exclusion criteria should be clearly defined in this section.

3. The selection of the patients without SOT should be defined in this section, and criteria for a propensity score match should be defined.

4. Study endpoints should be clearly stated.

5. Authors should refer to the selection criteria of the patients without SOT.

**Results**

1. The diabetes rate in Table 1 is misspelled.

2. After the propensity score match, the differences in covid-19 treatment should be explained.

3. Kaplan Meier analysis can be useful for determining differences in mortality between groups.

Patients with macrophage activation syndrome due to COVID-19 can be shown in the tables.

**Discussion**

1. The mortality rate of SOT recipients with COVID-19 was lower than in some of the previous articles. The authors must find out what factors can be associated with these differences.

2. The authors should discuss the impact of chronic kidney disease on covid 19 mortality in a separate paragraph.

3. Treatment modalities of the patients should be discussed in a separate paragraph.

4. The authors did not perform a power analysis before calculation, and the results may have been determined due to the second type of statistical error analysis. Therefore, authors should not use exact expressions.
