# Supplementary material for: A propensity score-matched analysis of mortality in solid organ transplant patients with COVID-19 compared to non-solid organ transplant patients
Source: PLoS One. 2021 Mar 3;16(3):e0247251. doi: 10.1371/journal.pone.0247251 (PMC7928439; doi:10.1371/journal.pone.0247251)
Supplement: Attachment — Submitted filename: PLOS one re-review.docx [file pone.0247251.s004.docx]

Dear editor,

Thank you for considering our revised-manuscript entitled “No increased risk of mortality in solid organ transplant patients with COVID-19”. Following your instructions we have addressed the new comments from reviewer 2 in a point-to-point fashion that we are attaching.

**Review Comments to the Author**

Reviewer #2: My concerns were below:

Thank you for your thorough review of our manuscript. We hope we have addressed all the concerns, and that the manuscript reads more clear now.

1) Title 
The title describes no increased risk of mortality, but authors must clearly define the group of the patient which, was shared the same risk of mortality between the solid organ recipient. I think that "No increased risk of mortality in solid organ transplant patients with COVID-19 compared to the non-solid organ transplant patients with COVID-19" or "A propensity score-matched analysis of mortality in solid organ transplant patients with COVID-19 compared to the non-solid organ transplant patients" may be better options.

*Authors: thank you for the suggestion. We have modified the title accordingly to: "A propensity score-matched analysis of mortality in solid organ transplant patients with COVID-19 compared to the non-solid organ transplant patients*".

2) Abstract

The authors defined the risk factors of mortality in solid organ transplant recipients. On the other hand, this issue is not written in this part. Authors should re-write or remove this sentence.
If this sentence is removed, criteria for a propensity score match would be useful for the readers.

*Authors: We have included your suggestion in the abstract.*

3) Introduction
Some of the major articles in this issue could not be referenced.
doi:10.1111/ajt.16185, 
doi:10.1056/NEJMc2011117, 
doi: 10.1097/TP.0000000000003533, 
doi:10.1097/TP.0000000000003433, 
doi:10.1111/ajt.16246, 
doi:10.1111/tid.13383, 
doi:10.1111/tid.13371, 
doi:10.1016/j.kint.2020.08.005 
maybe added to the references

*Authors: We have added the suggested references.*

Two sentences that began "The main objective" and "the aim of the study" were defined the same thing. The paragraph which began with "the main objective" may remove.

*Authors: We have deleted the paragraph that reviewer 2 mentioned.*

"Furthermore, besides immunosuppressive regimens, transplant recipients usually present more co-morbidities such as hypertension and diabetes, possibly influencing the outcome of patients with COVID-19. On the other hand, any propensity score match analysis was not performed in mortality of solid organ recipients compared to non-solid organ transplant patients before." may be a better option.

*Authors: we have replaced the paragraph as suggested by reviewer 2*

4) Material and methods
Serum may be added before the laboratory measurements; such as C-reactive protein, creatinine.

*Authors: we have added serum before the laboratory measurements.*

All patients were followed-up at least after discharge for two months.
There must be an anticoagulation and ventilation protocol for COVID-19 which, must be described in the material and method section.

*Authors: We have described the anticoagulation and ventilation protocol in the methods section.*

5) Results
The authors must describe why they measure in mean and SD for table 1 and median and IQR for table 2?

*Authors: We have changed data. All measures were calculated in median and IQR.*

Twenty-one patients were diagnosed with pulmonary emboli, does the patient screen for pulmonary emboli, or are they have any symptoms? This issue must be described in the material and method section.

*Authors: We have specified that only certain patients are screened for PE, ie: if there is clinical suspicion.*

Table 2 should be re-calculated after propensity score-matched analysis.

*Authors: In table 2 we compared the two groups before applying the propensity score-matched analysis. Comparison of transplant and non-transplant population after applying a propensity score analysis, using only variables we considered that could influence outcomes is shown in table 5.*

6) Discussion
These mortality differences might be explained by the recipient and donor age, donor type, and the number of co-morbid diseases.
One of the major limitations of the study, was the small sample size which, was not clearly described in this section. As I mentioned in the previous review, it may cause a type 2 error.

*Authors: We have highlighted in the limitations the small sample size might cause a typo 2 error.*
